# Modal Parameters Evaluation in a Full-Scale Aircraft Demonstrator under Different Environmental Conditions Using HS 3D-DIC

**DOI:** 10.3390/ma11020230

**Published:** 2018-02-02

**Authors:** Ángel Jesús Molina-Viedma, Elías López-Alba, Luis Felipe-Sesé, Francisco A. Díaz, Javier Rodríguez-Ahlquist, Manuel Iglesias-Vallejo

**Affiliations:** 1 Departamento de Ingeniería Mecánica y Minera, Campus Las Lagunillas, Universidad de Jaén, 23071 Jaén, Spain; elalba@ujaen.es (E.L.-A.), fdiaz@ujaen.es (F.A.D.); 2 Departamento de Ingeniería Mecánica y Minera, Campus Científico Tecnológico de Linares, Universidad de Jaén, 23700 Linares, Spain; lfelipe@ujaen.es; 3 Airbus Defence and Space, Paseo John Lennon s/n, 28906 Getafe, Spain; javier.i.rodriguez@airbus.com (J.R.-A.); manuel.iglesias@airbus.com (M.I.-V.)

**Keywords:** aerospace, full-field analysis, HS 3D-DIC, operational deflection shapes, natural frequencies, experimental modal analysis

## Abstract

In real aircraft structures the comfort and the occupational performance of crewmembers and passengers are affected by the presence of noise. In this sense, special attention is focused on mechanical and material design for isolation and vibration control. Experimental characterization and, in particular, experimental modal analysis, provides information for adequate cabin noise control. Traditional sensors employed in the aircraft industry for this purpose are invasive and provide a low spatial resolution. This paper presents a methodology for experimental modal characterization of a front fuselage full-scale demonstrator using high-speed 3D digital image correlation, which is non-invasive, ensuring that the structural response is unperturbed by the instrumentation mass. Specifically, full-field measurements on the passenger window area were conducted when the structure was excited using an electrodynamic shaker. The spectral analysis of the measured time-domain displacements made it possible to identify natural frequencies and full-field operational deflection shapes. Changes in the modal parameters due to cabin pressurization and the behavior of different local structural modifications were assessed using this methodology. The proposed full-field methodology allowed the characterization of relevant dynamic response patterns, complementing the capabilities provided by accelerometers.

## 1. Introduction

In the past decades many efforts have been made in the aerospace industry to control and reduce the noise level inside aircraft. In this sense, the selection of materials and design are critical issues regarding the mass, damping, and stiffness of the structure, which define the modal behavior and, hence, the noise. This issue involves different design tasks regarding active control and vibration absorbers [1,2], and also in the prediction by means of numerical models [3]. 

Experimental testing provides a fundamental feedback to improve the design and numerical models. One of the most interesting tests in transport applications is experimental modal analysis for identification of modal parameters [4]. This type of characterization is commonly performed using accelerometers. However, some sources of error are present in the measurements as a consequence of their invasive nature. Furthermore, a compromise is always required between the time and cost required for instrumentation and the resulting spatial resolution, especially in large structures. 

Recent progress in contactless full-field techniques provides the opportunity to extract substantially more information from realistic experiments. The benefit of being a non-invasive technique ensures that the structural dynamic response remains free of possible perturbations introduced by mass and damping of transducers and cabling. One of the most remarkable emerging techniques is laser Doppler vibrometry (LDV). It is a pointwise interferometric technique that performs velocity measurements in the laser beam direction. It can provide full-field information by sequential or continuous scanning [5,6]. This is typically carried out by an automated mirror that modifies the direction of the laser beam towards different points. Anthropomorphic robot arms are also employed to perform sequential measurements of objects with three-dimensional geometry by modifying the position and the orientation of the laser [7,8,9]. Digital image correlation is an optical technique for displacement and strain measurement [10]. With a stereoscopic system of high-speed cameras (HS 3D-DIC), this technique is able to provide dynamic information with higher spatial resolution in the three spatial directions using a simpler setup. In the case of LDV, the economic cost is noteworthy whether measurements in the three spatial directions are required. 

On the other hand, both accelerometers and LDV are provided with modern commercial software that deals with the signals and less time is required to post-process the captured information. Comparative information of HS 3D-DIC and LDV can be found in previous works [11,12,13]. The characterization of full-field mode shapes and operational deflection shapes (ODS) is one of the most interesting features using HS 3D-DIC. Sinusoidal signals whose frequency corresponds to a resonance of the system is employed for this purpose [11,14,15,16,17,18,19,20,21,22,23]. Besides, employing a broadband excitation instead of a fixed sine the full-field response of the specimen is registered, and frequency response functions (FRF) or other frequency domain transfer function are easily obtained. By analyzing the peaks that maximize the response it is possible to estimate the natural frequencies and the damping ratios [12,13,24,25,26,27,28,29,30,31]. Special interest presents those studies that employ image decomposition approaches [17,18,22,25,32,33] to reduce the full-field information to just a few shape descriptors [34,35,36,37]. In these works, it was demonstrated that HS 3D-DIC is valid for different materials, such as steel, aluminum, composites, and polymers. However, most of them analyzed simple elements, like beams or plate components, and in the case of real components, they were analyzed isolated from the final assembly.

**Figure 1:**
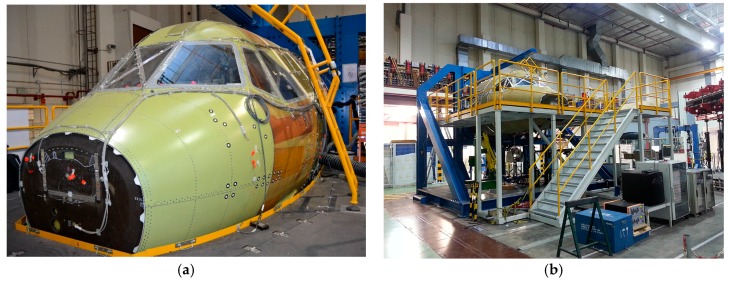
Clean Sky/Green Regional Aircraft MT2 cockpit demonstrator (**a**) and test rig (**b**) developed by Airbus Defence and Space.

In the work reported in the current paper, the HS 3D-DIC technique has been employed for the dynamic characterization of part of a front fuselage full-scale demonstrator (Figure 1) developed by Airbus Defence and Space in the frame of the Clean Sky/Green Regional Aircraft program, with partial funding by the European Union. For this purpose, measurements using HS 3D-DIC from the inside of the demonstrator have been conducted in the passenger window area, built using a multi-material component. This area of the cabin was considered relevant in the transmission of noise. The main purpose of these experiments was to characterize the specific vibration modes from measured displacements with major significance under a vibroacoustic perspective. In a previous work [38], results from sine and random excitation tests were explored and compared. Sine tests were employed for full-field ODS determination, while a random excitation test was employed to obtain the transfer function between the excitation and the window response in a full-field approach. From the transfer functions, both natural frequencies and full-field ODSs were identified. In the present work, this evaluation is extended. HS 3D-DIC has been also employed to evaluate the influence of in-flight conditions by applying a differential pressure to the demonstrator. Changes in the modal parameters were identified and it was found evidences of the influence in modal behavior of the deformation suffered due to pressure. Shape descriptor decomposition has been employed to enhance the managing of the full-field data and perform an analysis of the differences in the behavior of the window in those different tests. Finally, a second window specimen has been tested to study possible differences. Using this methodology it was possible to identify the variation on the dynamic behavior introduced by design. The results demonstrate the capabilities of the technique, providing new insights into the vibration behavior of a complex aerospace structure.

## 2. Three-Dimensional Digital Image Correlation

**Figure 2:**
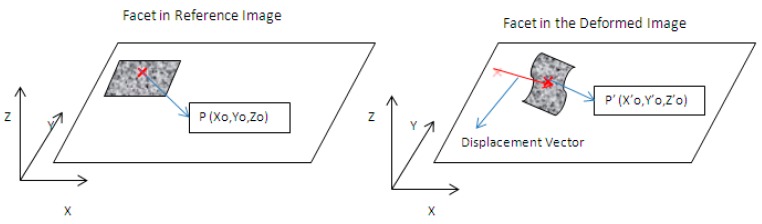
Displacement of a facet from a reference image to a strained image using DIC.

**Figure 3:**
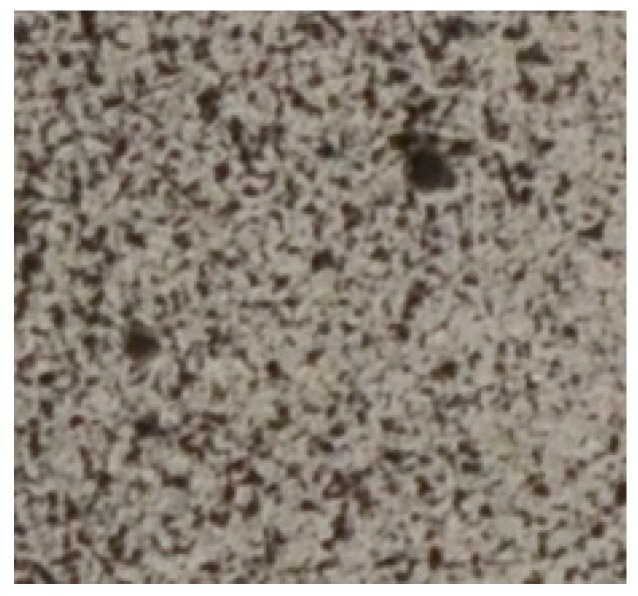
An example of a random speckle pattern.

Digital image correlation (DIC) is an optical technique used for measuring strain and displacements in mechanical elements [10]. In general terms, DIC correlates a sequence of digital images captured during the test and compares them with an image from an initial state, generally unloaded. The area of interest is virtually divided into squared regions known as facets. A facet is the smallest unit on which the algorithm performs tracking to infer the 3D displacements the specimen experienced and the associated strain field (Figure 2). This process is performed by matching the light intensity in the facet area corresponding to the initial and final position of each facet. In order to perform the tracking, every facet must be unique and, hence, a random speckle pattern, as shown in Figure 3, is needed in the area of interest.

**Figure 4:**
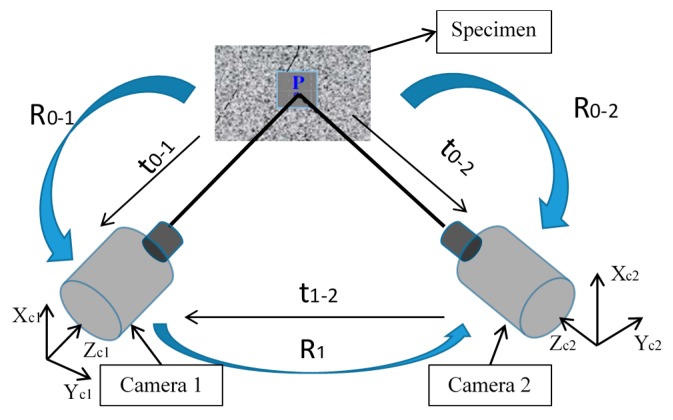
Calibration parameters in stereoscopic DIC system.

In 3D-DIC, stereoscopic images are required as well as system calibration based on triangulation of the data to obtain three-dimensional displacement measurements. In general terms, the implementation of the 3D-DIC technique requires the following steps:

1. Specimen and set-up preparation. The specimen must exhibit a random speckle distribution on its surface. Normally, it is coated with white paint and, subsequently, a random artificial speckle pattern is generated by spraying black paint over the white paint [39].
2. Calibration of the stereoscopy system. The calibration of the cameras is performed by placing a calibration plate with a printed grid on it in the test space, where the specimen is located during the experiment. Thus, it is possible to establish a correspondence with the local coordinate system of each camera and calibration parameters (Figure 4) [10,40,41,42].
3. Images capture. As was indicated above, a perfect synchronization of the cameras’ recording is necessary to correctly process the results. This is achieved by synchronizing the internal camera’s clock and simultaneous triggering.
4. Image processing. Images were processed to obtain the measured displacement and strain fields.

## 3. Materials and Method

Experiments were conducted using a full-scale composite cockpit demonstrator developed by Airbus DS in the frame of the Clean Sky Green Regional Aircraft program. Advanced materials and manufacturing technologies making extensive use of composites allowed a significant structural weight reduction. Multi-functional performance accounted for improved electric conductivity and lightning resistance, acoustic insulation, hail impact performance, and the use of embedded sensors for structural health monitoring.

This full-scale cockpit demonstrator was used for a comprehensive series of tests, including static and structural fatigue tests, impact damage tolerance, as well as vibroacoustic tests. It was in this context that the potential of HS 3D-DIC testing technology was evaluated for non-invasive structural dynamic testing, complementing conventional measurements using accelerometers. Measurements using HS 3D-DIC were performed in the window passenger area considering two different window specimens and the different types of tests hereafter described. Considering the number of tests and configurations, a summary is shown in Table 1.

### 3.1. Tests on Specimen 1

**Figure 5:**
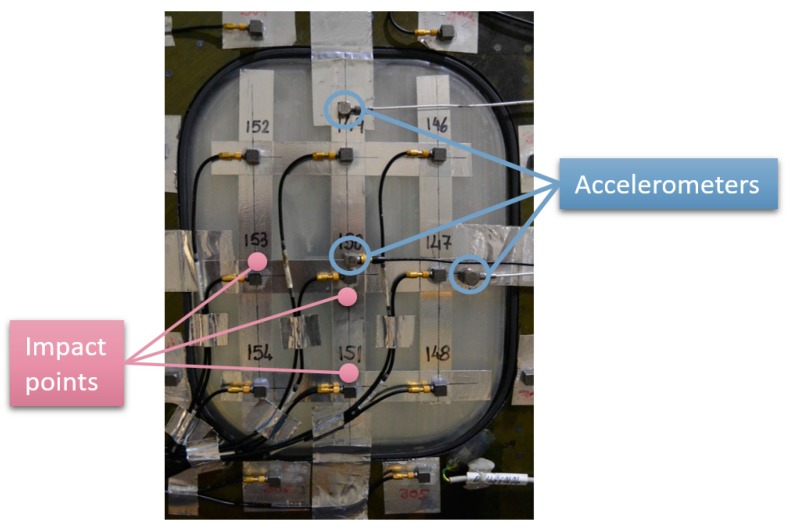
Set-up for impact hammer tests on the passenger window of the GRA front fuselage demonstrator (exterior view).

**Figure 6:**
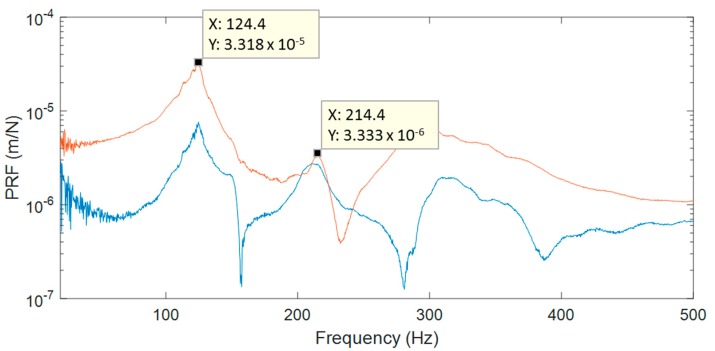
Two principal response functions from impact hammer tests of specimen 1.

Specimen 1 was employed to evaluate different issues with this methodology. Particularly, sine and random excitation signals were explored under atmospheric pressure. In sine tests, the structure was excited at a single frequency corresponding to a resonance and the ODS was obtained using HS 3D-DIC. Previous to HS 3D-DIC tests, an impact hammer test was performed to obtain a quick natural frequencies identification for the sine tests. A multi-input multi-output analysis was performed considering three measuring points and three excitation points. The location of the points was defined to obtain a reasonable characterization of the passenger window area, as shown in Figure 5. Non-highlighted accelerometers were intended for purposes unconnected with this study. Signals from the three accelerometers and the impact hammer were recorded by a Photon+ real-time analyzer (Bruel and Kjaer Sound & Vibration Measurement A/S, Nærum, Denmark). The FRFs were obtained by processing these signals considering a sampling frequency of 5120 Hz, 16,384 frequency lines, and 10 averaged windows. For the identification of the natural frequencies, the principal response functions (PRFs) were obtained through single-value decomposition of the FRFs matrix [4]. In Figure 6, two PRFs are shown and two significant frequencies are selected for sine tests. Afterwards, ODSs from the sine tests were compared with the same peak detected in the random test.

Additionally, the influence of in-flight conditions was evaluated by applying a differential pressure of 5.5 psi (34,473.8 Pa). Deformation due to pressure occurred in the whole demonstrator and, consequently, modifications of stiffness also occurred. Under these conditions, random excitation was applied in order to expose variations in the natural frequencies and ODSs.

### 3.2. Tests on Specimen 2

**Figure 7:**
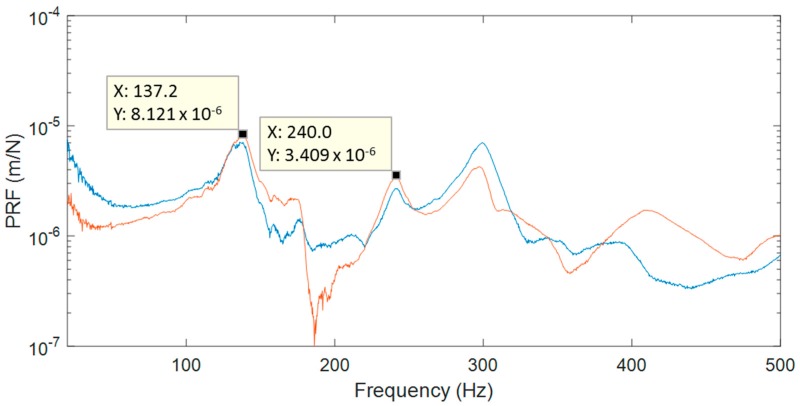
Two principal response functions from impact hammer tests of specimen 2.

With a second specimen, it was possible to evaluate the influence of a local structural reinforcement on the modal behavior of the passenger window area under atmospheric environmental conditions. Its behavior was studied using sine excitation, obtaining natural frequencies from impact tests and ODSs using HS 3D-DIC. The performance of the impact tests and identification of frequencies was as described for the first specimen. As highlighted in Figure 7, two significant modes were selected from PRFs at comparable frequencies to the first specimen. 

### 3.3. Test for Full-Field Measurement Using HS 3D-DIC

**Figure 8:**
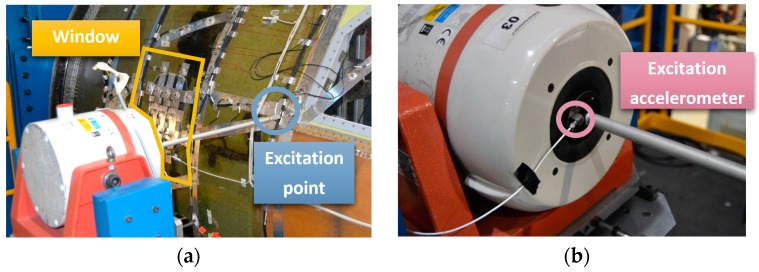
Lateral shaker excitation.

**Figure 9:**
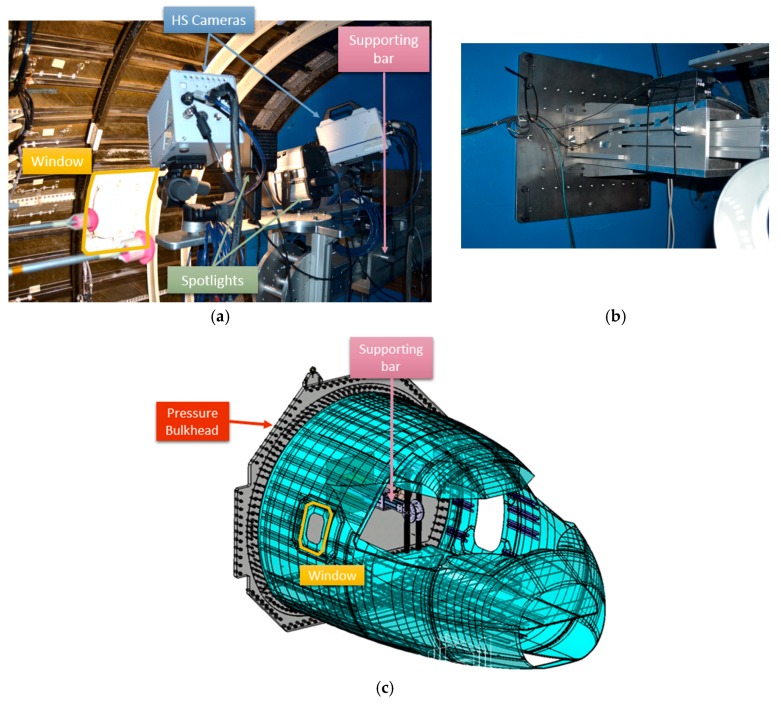
(**a**) Optical system on the supporting bar; (**b**) supporting bar attachment to the bulkhead; and (**c**) model of the global cockpit demonstrator configuration.

For sine and random excitation tests, an electrodynamic shaker model LDS V450 (311 N sine force peak, 214 N maximum random force RMS, and 5 Hz–7500 Hz frequency range) manufactured by Bruel and Kjaer Sound & Vibration Measurement A/S (Nærum, Denmark) excited the cockpit demonstrator laterally using a stinger, as shown in Figure 8a. HS 3D-DIC measurements were carried out on the window inner surface, i.e., inside the demonstrator. The set-up consisted in two, FASTCAM SA4 1024 × 1024 CMOS high speed cameras (Photron, Tokyo, Japan) with a maximum frame rate of 3600 fps at full resolution equipped with two AF Nikkor (Nikon Corporation, Tokyo, Japan) 50 mm f/1.4D lenses. Illumination is crucial, especially in high-speed image capturing. In order to achieve a uniform illumination without shadows or overexposed areas, the illumination system consisted of a Hedler DX 15 flood light (Cromalite S.L., Barcelona, Spain), and a Kaiser Videolight 6 (Kaiser Fototechnik GmbH & Co.KG, Buchen, Germany). As observed in Figure 9, the whole system was fixed in a unique structure specially designed for the proposed set-up. The rigid supporting bar was designed to be fixed to the pressure bulkhead (Figure 9b,c) in the structure that supports the whole demonstrator in order to avoid the mass effect of these elements on the demonstrator dynamic behavior, and to isolate the cameras from the excitation source. This support also provides four degrees of freedom to the optical system, making flexible and quick changes of configuration for measuring any part of the demonstrator possible.

**Figure 10:**
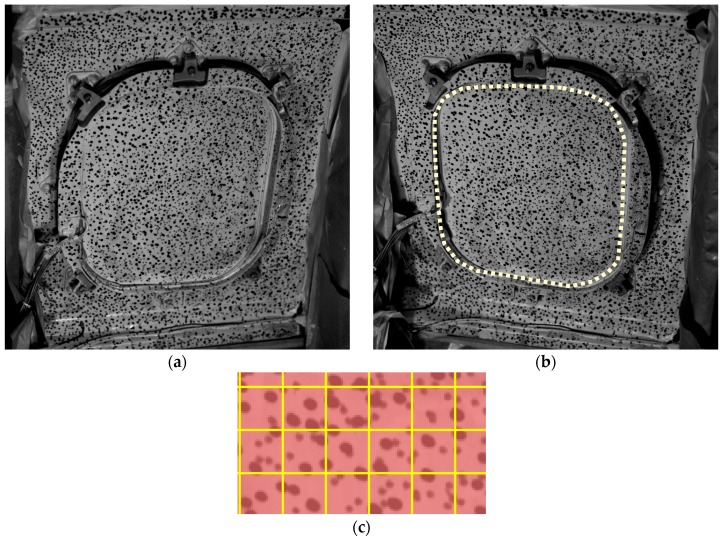
Images of the window from the cameras point of view. (**a**) Left-hand camera; and (**b**) right-hand camera. Dotted lines represent the area analyzed using shape descriptor image decomposition; (**c**) sample of the facets grid.

As observed in Figure 9a, the cameras were oriented to the window, which was randomly painted as shown in Figure 10. A commercial software package was employed for implementation of the 3D-DIC technique, namely Vic-3D (Correlated Solutions Inc., Irmo, SC, USA). This piece of software was employed for image processing and to obtain the in-plane and out-of-plane displacements. For DIC analysis, 19 pixels facets with a five-pixel overlap were employed, resulting in ca. 35,000 measurement points. In Figure 10c, some facets are shown as a grid to be visualized in comparison with the speckle size. In this study, out-of-plane displacements were employed since they define the bending deformation of the window. For the proposed configuration, the level of noise measured was 0.0079 mm. This value was determined as the RMS value of the displacements registered when processing two consecutive pairs of images during an unloaded state. 

The main requirement to determine ODSs during fixed sine tests was a good definition of the vibration cycle [43]. Thus, considering that the maximum frequency to characterize was 240 Hz, a camera frame rate of 2000 fps (frames per second) was employed, involving eight points per cycle.

During the tests where the demonstrator was excited using a random signal, it was possible to identify both natural frequencies and ODSs. This identification was performed from the full-field transfer functions between excitation and response. The excitation was monitored by an accelerometer on the shaker armature, shown in Figure 8b. The transfer function estimation required a perfect synchronization between the captured images (i.e., response displacements during the vibration) and the excitation signal from the accelerometer, thus, a NI USB-6251 DAQ system (National Instrument Corporation, Austin, TX, USA) was employed to allow simultaneous data collection. The natural frequencies were identified as the frequency where the resonance peaks occurs, and ODSs were obtained by the full-field depicting the imaginary part of the peaks for every single point. A spectrum from 0–640 Hz was employed for random excitation tests and the camera’s frame rate was set to 2000 fps, satisfying the Nyquist criterion. The spectral analysis was performed considering a frequency resolution of 1 Hz, anti-leakage Hanning windows, and an overlap of 50%.

### 3.4. Image Decomposition Comparison

The amount of experimental results for the two different kinds of tests was considerable. However, a comparison between the tests performed with fixed sine excitation and random excitation was required to validate the results using the latter type of test. For this purpose, an image decomposition method was employed. This method essentially consists on compressing large amounts of two-dimensional data by reducing its dimensionality to a feature vector while preserving the information [35].

This method has been widely employed for validation of analytical and theoretical models because it facilitates the comparison between 2D sets of data [34,35,36,37]. As commented, it consists of converting the information from different data maps (i.e., modal shape) obtained when the specimen is subjected to different conditions (or is analyzed employing different full-field techniques) into respective feature vectors composed by shape descriptors. This procedure is reversible, so, a 2D data field could be reconstructed from a feature vector. These feature vectors can be directly compared and are independent from original sized map or orientation [37]. The more similar the vectors, the more similar the results. The adopted image decomposition method was based on Chebyshev polynomials T_k_(i,j) to decompose displacement contours I(i,j) into a set of coefficients or shape descriptors, s_k_, with the same units as the original sets of data.

(1) $$I\left(i,j\right)=\sum_{k=0}^{N}s_{k}T_{k}\left(i,j\right),$$

The area of analysis using shapes descriptors were reduced to the transparent region, as highlighted in Figure 10b, where the largest displacements occur. 

**Figure 11:**
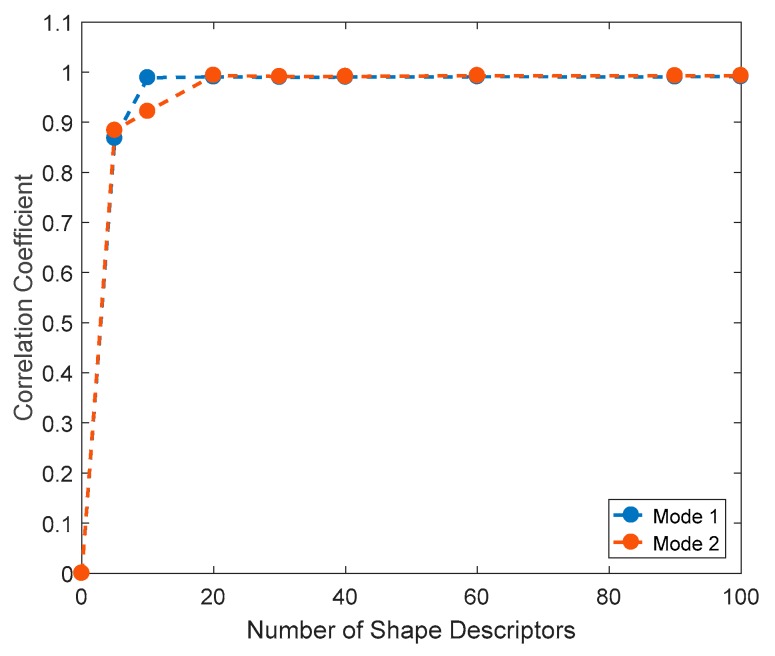
Evolution of the correlation coefficient between original and reconstructed displacement contours of specimen 1 as a function of the shape descriptors employed.

As mentioned, the feature vector should adequately preserve the information; specifically, the quality of the representation depends on the number of shape descriptors used for the image decomposition. This is evaluated by comparing the original data field to the one reconstructed employing a certain number of shape descriptors. In this case, the required number of shape descriptors was evaluated by analyzing the correlation coefficient between the original and the reconstructed image using different shape descriptors [37]. In Figure 11 the evolution of the correlation coefficient between original displacement maps and reconstructed map employing a certain number of shape descriptors is presented. Namely, blue and orange dots represent the correlation coefficient for Mode 1 and Mode 2 of specimen 1 from the sine tests, respectively. It is shown that the required number of shape descriptors to obtain a correlation coefficient above 90% is slightly different for each kind of displacement field compared. Similar results were obtained in the remaining tests. In this case, only 20 shape descriptors are sufficient to represent the displacement (ODSs) maps analyzed in this work.

## 4. Results and Discussion

**Figure 12:**
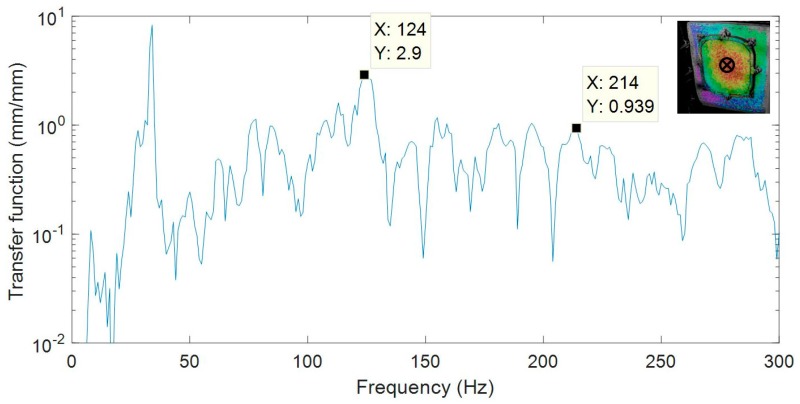
Transfer function of a point from the center of the window using HS 3D-DIC.

**Figure 13:**
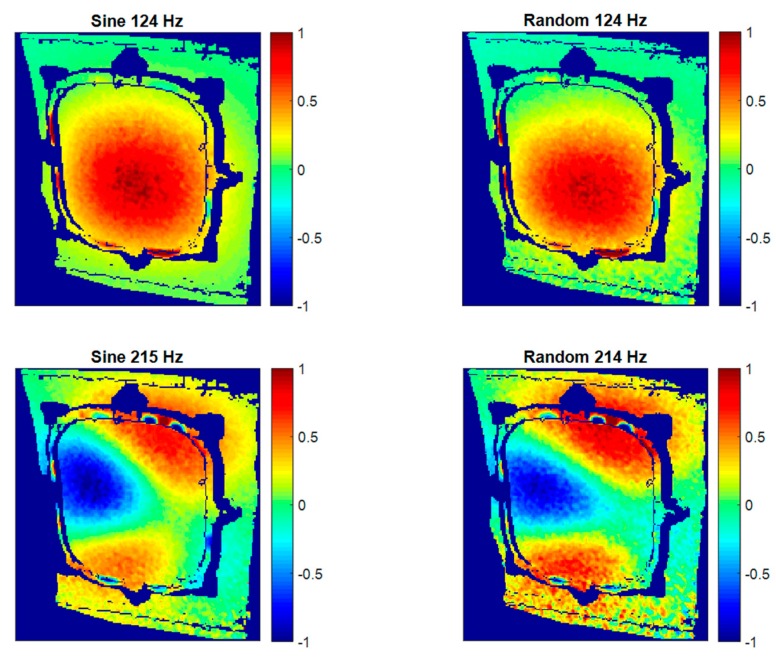
Normalized operational deflection shapes in the out-of-plane direction obtained using HS 3D-DIC under different excitation configurations.

In this section, modal identification using HS 3D-DIC in the described configurations is presented. Results of specimen 1 under an atmospheric environment is considered as a reference to discuss the results from the different configurations. The first results concern the evaluation of the modal parameters extracted from sine and random tests. From random, transfer functions, analogous to FRFs from impact hammer testing, are obtained. In Figure 12, the transfer function corresponding to a point in the center of the window is presented. This point was considered as representative of the main behavior of the whole window. A wide variety of modes are detected from the transfer function. Although excitation covered up to 640 Hz, for illustration purposes the transfer function is truncated at 300 Hz, considering that the most relevant modes for this study were below this limit. Two peaks are highlighted whose frequencies are close to those identified in the impact test. Consequently, these frequencies were considered to build ODSs. In the sine tests, ODSs were obtained directly from displacement measurements. Figure 13 shows the out-of-plane ODSs for each mode and test. They were normalized to enhance the visual and numerical comparison of the shape descriptors. 

Results in Figure 13 show a good agreement between sine and random tests. Numerically, the correlation coefficient between shape descriptors was 0.9803 for the first mode and 0.9593 for the second mode. In the first mode the window experiences a single bending with maximum displacement at the middle. The second one is in a more complex shape, as is expected from a higher-order mode, showing alternative bending regions. 

**Figure 14:**
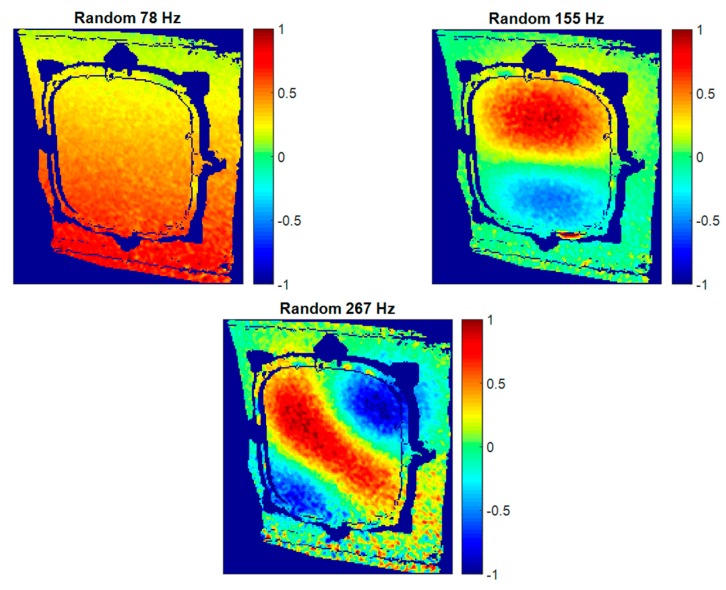
Additional normalized ODSs in the out-of-plane direction detected using HS 3D-DIC and random excitation.

Sine tests provide accurate, clearer results considering ODSs since the excitation energy is concentrated in just one single frequency, generating higher displacements. An additionally important point of these tests is that only a small number of images are required to describe some sine cycles. This number depends on the resonance frequency. Hence, the execution and processing times considerably diminish if a reasonable number of modes are characterized. However, sine tests are not able to provide information of resonance frequencies. It is necessary to previously know the natural frequencies’ values. Impact hammer tests using an accelerometer are a method to obtain this information. Moreover, in the particular cases here evaluated, since impact hammer tests only detect the local window’s resonances, the global behavior cannot be identified. Conversely, a broad spectrum is excited in random tests and it is possible to characterize as many ODSs as frequency lines resulted from the frequency analysis. More detailed information can be extracted from the structure, but it is also more time-consuming and ODSs could be slightly noisier, as seen in Figure 13. Some additional structural resonances detected using HS 3D-DIC and random shaker excitation are represented in Figure 14. They were selected according to their interesting ODSs. The resonance at 78 Hz corresponds to a global fuselage mode. As can be observed, this global mode appears locally as a rigid body motion since the analyzed frame is a small sample, considering the demonstrator size. Thus, the curvature of the mode is not perceived here. The other two ODSs at 155 Hz and 267 Hz imply local behavior and correspond to bending modes of increasing order of the window. Considering the high geometrical complexity of the presented ODSs, a non-negligible number of sensors would be necessary to properly characterize these shapes instead of HS 3D-DIC. Even so, the characterization would be sparse.

### 4.1. Specimen 1 under Differential Pressure

**Figure 15:**
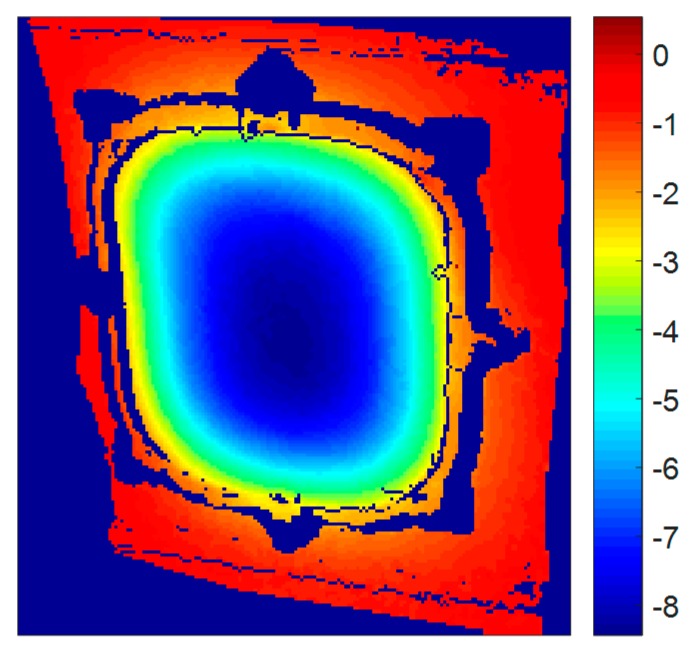
Static out-of-plane deformation (mm) of the window frame under differential pressure.

Differential pressure entails a homogenous force over the structure. In the passenger window area, the deformation due to differential pressure was measured by comparing the static state, shown in Figure 15. Maximum deformation occurs at the center of the window where the stiffness is lower. Deformations of more than 8 mm outwards are experienced in that particular point. This deformation modifies the shape of the window, increasing the curvature and, thus, providing a stiffer configuration. The stiffness increment of the structure is confirmed in the resonant frequency shifting towards higher frequencies. For the local resonances identified during random tests, Table 2 shows the frequencies corresponding to both states. 

**Figure 16:**
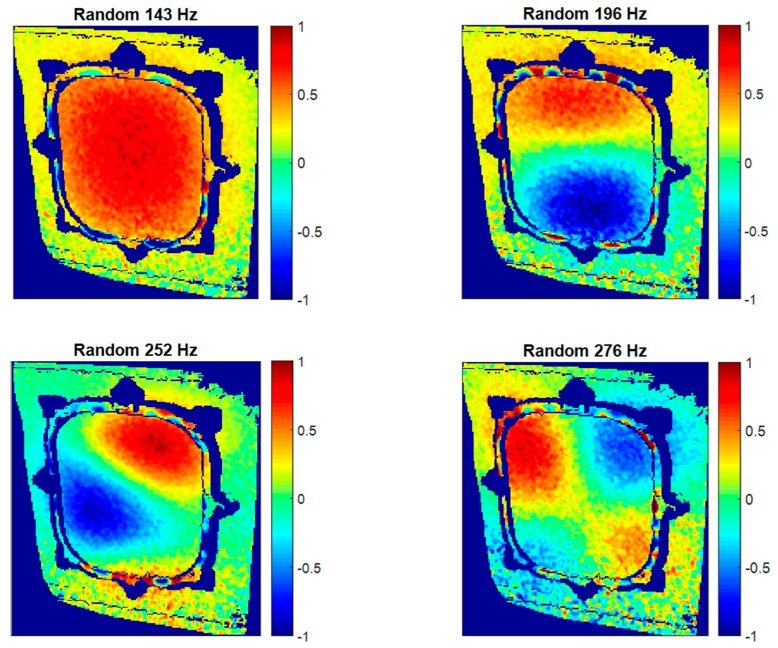
Normalized ODSs in the out-of-plane direction obtained using HS 3D-DIC in a random excitation test under differential pressure.

**Figure 17:**
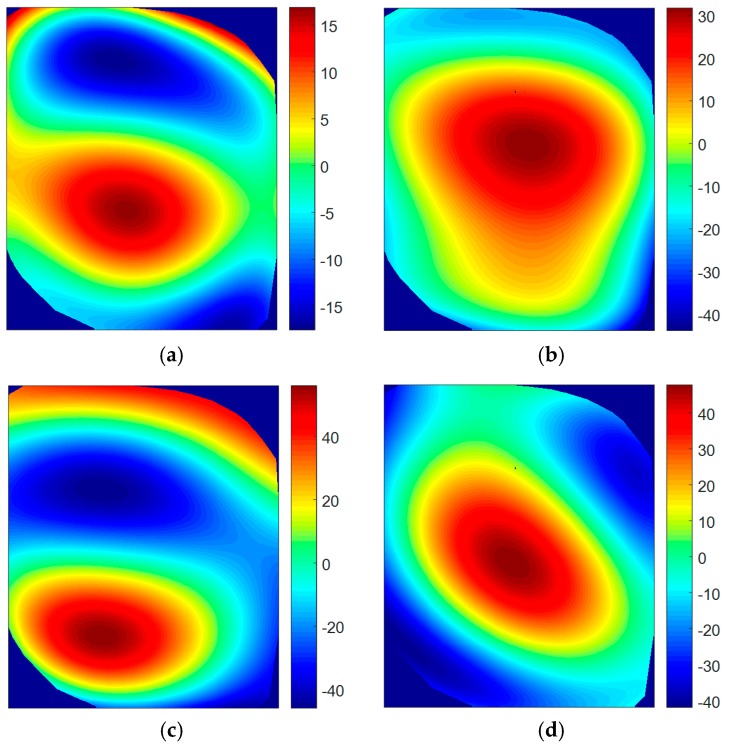
Reconstruction of the percentage differences between shape descriptors representing both ODSs in non-pressurized and pressurized states. (**a**) Mode 1; (**b**) Mode 2; (**c**) Mode 3; and (**d**) Mode 4.

Shapes, shown in Figure 16, confirm the correspondence of the modes with respect to non-pressurized results, although slight differences are noticed. These differences are evaluated looking at the variations in the shape descriptors for every mode obtained by the image decomposition method. In this case, differences were visualized by reconstructing the corresponding difference vectors for Mode 1, 2, 3, and 4. These were obtained by subtracting the feature vectors that describe the non-pressurized and pressurized states. Results of these differences are presented as percentages in Figure 17, considering the non-pressurized state as a reference. The first three shape descriptors were omitted since they represent rigid body translation and rotation [22]. Hence, the focus was placed on the local deformation of the window. A common behavior is noticed in the ODSs. Maximum positive difference is located in the center of the window, where maximum static deformation due to pressure was found. This means that this region experienced more deformation with no differential pressure. Thus, the correlation between variations in ODSs and the pressure deformation is shown. This phenomenon is not detected in the third mode (Figure 17c) as a consequence of the non-symmetry of the shape. Considering that the shape of the first resonance is similar to the deformation due to differential pressure, the lowest differences are found in this ODS.

### 4.2. Specimen 2

**Figure 18:**
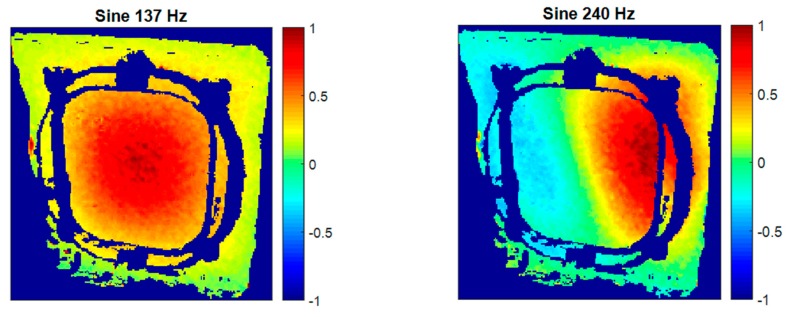
Operational deflection shapes of the second window specimen obtained using HS 3D-DIC in a sine test.

As a result of evaluating HS 3D-DIC measurements in the second specimen in a sine test at the previously-identified frequencies, two ODSs were characterized, shown in Figure 18. As can be observed, the ODS at 137 Hz is equivalent to that in the first specimen at 124 Hz. However, the frequency is higher in this specimen as a consequence of an increment in the stiffness. The ODS at 240 Hz is exclusive of this specimen. As it can be seen, the window experiences positive displacements in the right side, and negative on the left, with zero displacement in the middle. This is, thus, a second-order mode. In comparison with the ODS measured at 214 Hz in specimen 1, this is a lower-order mode, but at a higher frequency. Consequently, resonances were shifted to higher frequencies with this specimen and stiffening is confirmed.

## 5. Conclusions

In the present paper a full-field methodology for modal identification of aircraft structures using HS 3D-DIC has been presented. Focus was placed on the local dynamic response of the passenger window area, namely, natural frequencies and full-field ODSs. Shape descriptor decomposition was employed to ease the managing and enhance the analysis of the full-field data. 

Results presented in this document demonstrate the potential of HS 3D-DIC for different excitations, such as sine and random excitations. Sine excitation was less time-consuming when a low number of ODSs are required and provided less noisy results. However, previous knowledge of the natural frequencies is required. Conversely, random excitation allows the analysis of the behavior in a broad spectrum in a unique test. Full-field transfer functions from random tests allowed both the identification of resonances and the extraction of ODSs. 

The effect of a local structural modification was evaluated at different cabin pressure conditions. Stiffening as a result of the pressure deformation was identified in the resonance shifting and in the variation of the ODSs. The analysis of this difference using shape descriptors decomposition showed a common trend related to the deformation due to differential pressure.

The methodology was also able to provide information about different specimens maintaining the optical set-up. Variations in the natural frequencies and in the mode shapes were detected. This information allows to experimentally evaluate the behavior attending to the design and materials with more detailed information than traditional sensor instrumentation. 

This makes HS 3D-DIC interesting for contactless structural dynamic characterization of complex structures with no alteration of the behavior, and particularly for the purpose of comparison with numerical simulation. A window is mounted in a full-scale demonstrator, hence, all results correspond to the final behavior and the influence of the cabin deformation over the window behavior was reported.

## Figures and Tables

**Table 1:** Summary of the test configurations.

Excitation	Sensor	Specimens	Environment	Results
Impact	Accelerometers	1 and 2	Ambient	Natural frequencies
Sine	HS 3D-DIC	1 and 2	Ambient	ODSs
Random	HS 3D-DIC	1	Ambient and differential pressure	Natural frequencies and ODSs

**Table 2:** Frequency shifting of the resonances identified in both pressurized states in specimen 1.

Mode Denomination	Non-Pressurized (Hz)	Differential Pressure (Hz)
1	124	148
2	155	196
3	215	252
4	267	276
